# Deep learning-based pupil model predicts time and spectral dependent light responses

**DOI:** 10.1038/s41598-020-79908-5

**Published:** 2021-01-12

**Authors:** Babak Zandi, Tran Quoc Khanh

**Affiliations:** Department of Electrical Engineering and Information Technology, Laboratory of Lighting Technology, Technical University of Darmstadt, 64289 Darmstadt, Germany

**Keywords:** Visual system, Human behaviour

## Abstract

Although research has made significant findings in the neurophysiological process behind the pupillary light reflex, the temporal prediction of the pupil diameter triggered by polychromatic or chromatic stimulus spectra is still not possible. State of the art pupil models rested in estimating a static diameter at the equilibrium-state for spectra along the Planckian locus. Neither the temporal receptor-weighting nor the spectral-dependent adaptation behaviour of the afferent pupil control path is mapped in such functions. Here we propose a deep learning-driven concept of a pupil model, which reconstructs the pupil’s time course either from photometric and colourimetric or receptor-based stimulus quantities. By merging feed-forward neural networks with a biomechanical differential equation, we predict the temporal pupil light response with a mean absolute error below 0.1 mm from polychromatic (2007 $\pm$ 1 K, 4983 $\pm$ 3 K, 10,138 $\pm$ 22 K) and chromatic spectra (450 nm, 530 nm, 610 nm, 660 nm) at 100.01 ± 0.25 cd/m^2^. This non-parametric and self-learning concept could open the door to a generalized description of the pupil behaviour.

## Introduction

The development of a generalized human pupil model, which is able to predict the pupil aperture depending on photometric or physical quantities, has not been finished. Starting with the first pupil studies by Blanchard^1^ and Reeves^2^ in 1918, after more than 100 years of research, no valid model has been developed that summarizes the pupil control path's essential dependencies. Since the discovery of the intrinsically photosensitive retinal ganglion cells (ipRGCs), research has mainly focused on the understanding of the neurophysiological process behind the pupil light reflex but less on summarizing this outcome in a combined model. Even before the ipRGC-turning-point, it is noticeable that the parameters of time and wavelength dependence, including the chromatic adaptation effect, were not considered in the development of pupil models.

Clyde Keeler showed in 1926 that blind and rod-less mice still exhibited a persistent pupillary light response^3^. He indicated that a part of the afferent pupil path is controlled by a mechanism which might be independent of vision^3^. Studies with monochromatic light stimuli confirmed Keeler’s hypothesis by showing that the pupil light response's wavelength sensitivity cannot be described by the photopic luminous efficiency function V(λ) alone^4–6^. The pupil light reflex's wavelength sensitivity has a temporal influence, exhibiting a shift of the peak sensitivity with increasing adaptation time from 510 nm to the short wavelength range of 470 nm^7,8^. Such an effect could only be explained after the discovery of intrinsically photosensitive ganglion cells in the retina^9,10^, which have a peak sensitivity of approximately 470–480 nm.

Six different subtypes of ipRGCs (M1–M6)^11,12^, project to the olivary pretectal nucleus^13,14^, the dorsal lateral geniculate nucleus^15–17^, and the suprachiasmatic nucleus of the hypotalamus^18,19^. The M1-ipRGCs are part of the afferent control path, responsible for the sustained constriction mechanism through the olivary pretectal nucleus and the Edinger-Westphal nucleus^9,20^. In the inner retina, M1-ipRGC dendrites receive and integrate extrinsic synaptic signals^21^ from rods, with an additive contribution of L- and M-cones and separate inhibitory input from S-cones^10,11,22–30^. Additionally, investigations with sinusoidal or rectangular modulated stimuli showed an inhibitory contribution of M-cones^31,32^ and influences from the parvocellular pathway with chromatic red-green signals, which might be a post receptoral mechanism^33–35^. Depending on which spectral, spatial and temporal stimulus modality is used, the receptors are weighted differently in controlling the afferent pupil control path.

At photopic adaptation with a steady-state light stimulus, the outer classical photoreceptors manage the phasic pupil diameter, while the ipRGCs dominate the tonic pupil diameter^36–38^. Up to the equilibrium state of the pupil, the weighting proportion of the classical outer retinal photoreceptors and the ipRGCs is time dependent^38^. The period at which the equilibrium state is reached depends significantly on the retinal irradiance and the spectral power distribution of the stimulus^7,25,39,40^. Pupil examinations showed that the equilibrium state is reached faster with short-wavelength stimuli than with longer-wavelengths^25,39,41^. Thus, a distinction must be made between a phasic and a tonic pupil light response.

Historically, these notable findings have had little impact on pupil modelling research. The origin of pupil modelling begun with the functions of Holladay^42^ and Crawford^43^, each based on investigations of unknown age. With their ground-breaking works, they set the requirements for upcoming pupil models; developing a model that can predict the pupil diameter as a function of a V(λ) weighted quantity. It was indirectly assumed that the pupil control path is managed by an additive combination of L- and M-cones. This assumption is the basis of all published pupil models until the year 2012. Moon and Spencer^44^ and De Groot and Gebhard^45^ created combined models based on previously published data sets. These two models differ mainly in the predicted pupil diameter at high and low luminance. The models from Crawford and Moon and Spencer both used a hyperbolic tangent fitting function, taking care of the minimum and maximum pupil diameter. De Groot and Gebhard^45^ believed that an intense saturation of the pupil diameter at high luminance using a hyperbolic tangent function does not correspond to the pupil’s physiological nature. However, a high raw data variance between all authors up to the year 1952 is noticeable, which is justified by Stanley and Davies^46^ with differently sized adaptation surfaces. Therefore, Stanley and Davies^46^ proposed a pupil model that integrates the adaptation field size as an additional dependent parameter. Watson and Yellot^47^ reviewed all pupil formulas and developed an unified pupil model with the additional parameters “age” and “number of eyes”. Including the model from Watson and Yellot^47^, all formulas predict the static sustained pupil diameter in millimetres at the equilibrium state, caused by white light from thermal radiators. The time and spectral dependency of the afferent pupil control path were not taken into account in any of these models, although these are essential dependence parameters. In 2017, Rao et al*.* published a pupil model that takes into account the influence of ipRGCs by using a cirtopic luminance as an additional parameter^48^. The model was based on pupil examination, which used white light from phosphor-converted LEDs with an exposure time of 80 s. However, using the model requires knowledge about the measured stimulus spectrum, which complicates its application compared to L- and M-cone based pupil models. Therefore, the more rigorous application must make a significant contribution to the prediction accuracy, justifying the extra work. In a recent study, it was found that at 60 s exposure time, the mean prediction error of the Watson and Yellot pupil model with polychromatic white light of different correlated colour temperatures ($\sim$ 2000 K, $\sim$ 5000 K, $\sim$ 10,000 K) is less than $\pm$ 0.5 mm^25^. At one second exposure time, it was 0.71 ± SD 0.15 mm^25^. Furthermore, with chromatic spectra of the peak-wavelengths 450 nm, 530 nm, 610 nm and 660 nm, the averaged prediction error at one second adaptation time was 0.94 ± SD 0.12 mm^25^.

Therefore, adding a static ipRGC-component for the steady-state pupil diameter for longer exposure times like in the model from Rao et al*.* is not sufficient. The temporal influence is much more significant than the spectral impact when using white polychromatic light spectra^25^.

Neither the dynamic receptor weighting nor a time-dependent prediction of pupil diameter is possible with any state-of-the-art pupil model. Even with spectra along the Planckian locus, pupil models reveal flawed predictions due to the missing time dependence, showing that being able to reconstruct the wavelength-dependent time course of the pupil light response would be the next step^25^. Moreover, the history of pupil modelling showed that parametric model approaches with fixed functions are not sustainable. When adding additional dependent parameters or renewing the data, the whole structure of the model has to be changed. With this work, we aim for a non-parametric and data-driven model approach, which can consider additional stimulus dependencies without changing the model structure itself. This could make it possible to build a self-learning pupil model based on a publicly accessible database, leading to a general pupil behaviour function. The published standards in pupil research have created a basis for the vision of such a pupil light database^49^.

Here, we developed a concept for a deep learning-based pupil model that can consider the temporal and adaptive weighting dependence of the retinal receptors. We combined time-variant and time-invariant model approaches with a data-driven non-parametric neural network to link model parameters with spectral stimulus quantities, making it possible of reconstructing the pupil light response up to its' equilibrium-state by using only photometric and colourimetric, or receptor-based stimulus quantities.

## Materials and methods

### The requirements for a time- and wavelength-dependent pupil model approach

The structure of state-of-the-art pupil model approaches needs to be changed when additional exogenous influencing parameters inside the function are necessary. For instance, the age of subjects $y$ significantly affects the pupil diameter ${d}_{p}$, because the maximum aperture decreases with rising age^50^. To take this achromatic effect into account, Watson and Yellot had to modify the function of Stanley and Davies by embedding it into another function to derive the age dependency $y$ in the unified pupil model ${d}_{p,Watson}(L, y, e,\alpha )$. Such a strategy is not effective and would not have been necessary for a data-driven non-parametric pupil model.

Given the pupil's dependency parameters, it is foreseeable that cognitive influences will be included to improve the prediction accuracy in the future. Such cognitive influencing parameters can cause intersubject or intrasubject scatter in the measured raw data. Studies have shown that the intrasubject variance of a single participant reaches from $\pm$ 0.3 mm to $\pm$ 0.6 mm^51,52^. A higher variance of up to ± 1.5 mm^43,50^ is associated with intersubject studies^43,50^. Thus, a pupil model can never be more accurate than these variances. Large sample sizes behind a pupil model lead to an improved model quality since the mean of the population is approximated more accurately. A generalised pupil model would not actively decrease the prediction error of a single observer. However, by knowing the pupil diameter’s distribution of a population at a given stimulus, a confidence measure could be modelled too.

Non-parametric functions that have sufficient degrees of freedom are the key to make a data-driven model possible. Before cognitive influences can be modelled, an approach must be found to model the complex properties of the exogenous influences to the afferent pupil path. In this area, there is a gap that has not been closed.

**Figure 1:**
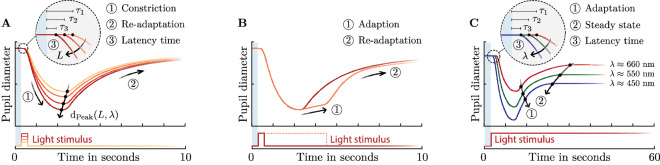
Idealized representation of the pupillary light response caused by different types of stimuli. (**A**) Phasic pupil response at one short light pulse. As the luminance intensity increases, the constriction velocity and the peak pupil constriction increases. At the same time, the latency of the pupil decreases with increasing luminance intensity. After peak constriction, the pupil undergoes re-adaptation. (**B**) During longer exposure times, the pupil adapts to the light stimulus itself, resulting in an increasing dilatation up to the equilibrium state. (**C**) The latency time, constriction velocity and peak constriction depend on the used light wavelength. Short wavelength stimuli cause a lower latency and a greater pupil constriction. The equilibrium time is reached faster with short wavelengths.

The afferent pupil path's mechanism affects the temporal constriction and dilatation of the pupil differently depending on the radiance ${L}_{e,\Omega }$ of the stimulus spectrum $x(\lambda )$ for $\lambda \epsilon$[380, 780], and exposure time ${t}_{L}$. When using short exposure times ($0<{t}_{L}\le 2$ seconds), the pupil reacts after a latency time $\tau$ of 220 ms to 550 ms and contracts up to a peak^53^ diameter ${d}_{Peak}$, followed by a re-adaptation phase in which the pupil diameter dilates back to its pre-stimulus state (Fig. 1A).

When a stimulus spectrum $x(\lambda )$ is constant, the latency $\tau$, constriction velocity and peak constriction depends on the used radiance ${L}_{e,\Omega }$ or luminance $L$ of the light source. As the luminance $L$ increases, the constriction velocity and peak constriction increase while the latency time $\tau$ reduces^53–55^ (Fig. 1A). The afferent pupil control path starts adapting to the stimulus $x(\lambda )$ itself after the peak constriction when the exposure time of the stimulus ${t}_{L}$ is increased. In this adaptation phase, the influence of the L-, M- and S-cones decreases and the melanopsin-activated ipRGC signal reaches its dominance^7^. This adaptive weighting of the receptors causes the decrease ("pupil escape") of the initial peak constriction with increasing adaptation time (Fig. 1B). When steady-state light stimuli with constant luminance $L$ but different chromatic spectra $x(\lambda )$ are used, the pupil light response's wavelength dependency becomes more apparent. Studies have shown that both the latency time $\tau$ and the peak constriction ${d}_{Peak}(L, \lambda )$ are wavelength dependent. The pupil contracts stronger and faster at short wavelengths than at long wavelengths^36,56–58^. Additionally, the chromatic pupil adaptation mechanism at longer wavelengths takes more time to reach the equilibrium state^39,59,60^ (Fig. 1C). Therefore, the pupil light response can be defined as ${d}_{p}(t, x(\lambda ))$. Existing L- and M-cone based pupil models only predict a static pupil diameter ${d}_{p}(L)$ with the luminance $L$ at the equilibrium-state.

Neurophysiological or practical models derived from empirical data are conceivable to describe these time- and wavelength-dependent processes. The neurophysiological approach would have the goal of deriving the photons to photoreceptor relationships all the way up to the transmission of frequency-coded action potentials via the afferent pupil path and the regulation of the iris muscles by the Edinger-Westphal nucleus, allowing to reconstruct the complex temporal pupil responses (Fig. 1A–C). Although such an approach would have the advantage of modelling the neurophysiological findings in recent years, it would make its application considerably more difficult for the latter, since knowledge of the spectrum and calculated receptor signals would be the prerequisite. It must be taken into account that the prediction of L- and M-cone based pupil models are flawed, but often used, since they can calculate the pupil diameter by using standard measurement equipment. Therefore, an alternative pupil model must be able to compensate for the deficits of current L- and M-cone models and give the possibility of adding additional model dependencies.

### Participants

We used the data from an intra- and intersubject pupil experiment with chromatic and polychromatic spectra to develop and train the proposed data-driven pupil model approach^25^. The complete pupil data used in this manuscript are from the authors’ previous publication^25^. Therefore, the methodology in the collection and pre-processing of the participants’ data is reported from the previously conducted experiments^25^. The pupil experiments were split into a chromatic and polychromatic stimuli session. The subjects in the chromatic trial had an age between 19 to 25 y, mean age 21.95 SD $\pm$ 1.73 y. In the chromatic session, the observers were 19 to 25 years old, mean age 22.2 SD $\pm$ 1.77 y. One subject was tested in-depth with twelve repetitions (Age: 33 y). Participation’s prerequisite was an age range between 19 to 25 y, no history of ocular disease, no use of medications or drugs that could influence the pupil response. Furthermore, we instructed the subjects to drink no caffeine and alcohol 48 h before the experiment. The study was approved by the ethics committee of the Technical University of Darmstadt (ID: EK 12/2019) and carried out in accordance with the ethical principles of the Declaration of Helsinki^25^. All guidelines and regulations of the TU Darmstadt’s ethics committee were met. We have received a signed consent from all participants.

### Photometric setup conditions and experimental protocol

The stimulus spectra were generated using an active temperature-controlled 15-channel LED light^25^. Eleven LED channels were narrow-band light-emitting diodes with the peak wavelengths 420 nm, 450 nm, 470 nm, 505 nm, 530 nm, 545 nm, 590 nm, 610 nm, 630 nm, 660 nm, 720 nm and full widths at half maximum were 14 nm, 18 nm, 25 nm, 29 nm, 33 nm, 105 nm, 78 nm, 17 nm, 16 nm, 17 nm, 29 nm. Four channels consisted of phosphor-converted white light-emitting diodes with correlated colour temperatures of 2700 K, 4000 K, 5000 K and 5500 K. The LED-circuit boards were regulated to a temperature of 30 $\pm$ 0.1 °C. Flicker effects were avoided by setting the PWM-frequency to 2 kHz. The luminaire was placed on top of an observation chamber to mix the rays inside the experimental box^25^. Through a mirror inside the box, a homogeneous illuminated 700 × 700 mm rectangular surface was reached, corresponding to a visual angle of 53.1°. The gaze position was fixed to the middle of the adaptation surface through a 0.8° fixation target from Thaler et al., consisting of a bull-eye combination with a cross-hair structure^61^.

The pupil measurements from the authors’ previous publication to obtain the training data were split into two studies^25^. In the first study, chromatic LED spectra with the peak wavelengths 450 nm (99.73 SD $\pm$ 0.4 cd/m^2^), 530 nm (100.12 SD $\pm$ 0.2 cd/m^2^), 610 nm (100.16 SD $\pm$ 0.2 cd/m^2^) and 660 nm (99.97 SD $\pm$ 0.2 cd/m^2^) were used. The second study was conducted with polychromatic spectra along the Planckian locus with correlated colour temperatures of 10,138 SD $\pm$ 22 K (99.83 SD $\pm$ 0.2 cd/m^2^), 4983 SD $\pm$ 3 K (100.10 SD $\pm$ 0.4 cd/m^2^) and 2007 SD $\pm$ 1 K (100.17 SD $\pm$ 0.3 cd/m^2^). For simplicity, we labelled these spectra as $\sim$ 10,000 K, $\sim$ 5000 K and $\sim$ 2000 K. The Polychromatic spectra were optimized using a heuristic multi-objective optimization method (genetic algorithm). On each experimental day, the spectra were measured twenty times using a calibrated Konica Minolta CS2000 spectroradiometer. The spectra are reported in the Supplementary Table S2.

Within the experiment, the stimuli were presented in a fully randomized order, each with 300 s adaptation time. The longer adaptation time was intended to capture the pupil light response up to its’ equilibrium state, ensuring that our model approach had training data for the complete pupil adaptation^8^. Prior to each stimulus, a reference stimulus of 5500 K (199.45 SD $\pm$ 0.43 cd/m^2^) was switched on for 300 s to adapt the pupil back to a baseline. The luminance increment between the anchor and stimulus spectrum was intended to provide a comfortable transition between the chromatic and phosphor-converted anchor spectrum^25^. Preliminary studies showed that at steady luminance the transition between the anchor and 450 nm spectrum was uncomfortable for the subjects, leading to increased eye blink rates in phasic pupil data^25^. For comparability, the anchor luminance was preserved in the second study with polychromatic spectra.

One test session took 40 min with the chromatic spectra and 30 min with the polychromatic stimuli. The observers fixed the target inside the observation chamber during the whole time, to avoid pupil foreshortening error^62^. An instructor checked the gaze position of the participants with real-time gaze tracking.

### Pupil measurement and pre-processing of the data

The pupil diameter of the left eye was recorded during the whole 300 s adaptation time with an extrinsic and intrinsic-calibrated stereo camera system at 120 frames/s from Smart Eye Pro, consisting of two 659 × 494 pixels Basler acA640-120gm cameras and 8 mm lenses. Camera calibration was performed with a checkerboard, resulting in an average accuracy of 0.15 mm for edge detection. Prior to each experiment, gaze calibration was conducted with the participants. We removed the blink-artefacts from the pupil data with the blink detection algorithm from Smart eye pro. All pupil data which had an edge detection accuracy less than 97 percent were deleted from the dataset. Other non-physiological artefacts were cleaned by using a velocity filter. The pupil data were differentiated numerically and all strong outliers with a percentile threshold criterion of 99.993 and 0.007 percent were removed. We linearly interpolated all missing data. The pupil data were smoothed using a Savitzky-Golay-Filter with a window size of 3000 data points. However, the first three seconds were excluded from the smoothing, to avoid artificially induced minimization of phasic pupil diameter.

### The concept of modelling the pupil light response

Our empirical modelling approach of the time- and wavelength-dependent pupil light response aims to reconstruct the pupil diameter using the respective photometric and colourimetric parameters from which it was triggered. There is a direct and indirect approach to this task. The direct way would be to train a recurrent neural network with measured empirically collected pupil data ${d}_{p,meas}({t}_{1}, {t}_{2},\dots , {t}_{n})$ for ${t}_{1}, {t}_{2},\dots , {t}_{n} \epsilon {\mathbb{R}}^{C}$ with $C$ for each stimulus condition. When designing the neural network, the input parameters (features) would be a sequenced abstraction $\{{x}_{i}\}_{i=1}^{N}$ ${x}_{i} \epsilon {\mathbb{R}}$ of the stimuli spectrum and the output ${d}_{p,out}({t}_{1}, {t}_{2},\dots , {t}_{n})$ would be the pupil diameter per time unit $t$. The number of input parameter $N$ could be chosen freely, but its goal is to provide enough information, allowing the neural network to reconstruct the pupil diameter ${d}_{p,out}(t)$. For instance, it would be possible to use different combinations of luminance, CIExy-2° chromaticity coordinates and receptor signals as input values ${\{{x}_{i}\}}_{i=1}^{N}$. The combination of luminance and CIExy-2° chromaticity with coordinates ($N$ = 3) would have the advantage of considerably simplifying the use of the later model since the knowledge of a spectrum is not required to predict the pupil light response ${d}_{p, out}(t)$. Usually, sequence-to-sequence recurrent neural network architectures are used for such tasks, but they require a substantial amount of data to achieve the desired accuracy. The accuracy would be limited by the skew of the number of parameters ($N, n$) between input and output. At a resolution of one second with $t \epsilon \left[0, 300\right]$, the neural network output would correspond to 300 pupil diameter values, which needs to be determined from three photometric quantities (L, CIExy-2°) as input $\{{x}_{i}\}_{i=1}^{N=3}$. Even if the time resolution of the set is halved and the number input parameters $N$ increased, a neural network would still have to determine 150 diameter values ${d}_{p,out}(t)$ values from six input values $\{{x}_{i}\}_{i=1}^{N=6}$ (CIExy-2°, luminance, L-cone, M-cone, S-cone, melanopsin signal). The reconstructed pupil data should not exceed a mean absolute error of $\sim$ 0.5 mm, since existing L- and M-cone models already predict the polychromatic spectra caused pupil diameter in such an error range^25^. However, for today’s pupil research applications, a model’s prediction error should not exceed $\sim$ 0.1 mm as cognitive and vision science focuses on smaller diameter margins^63^.

For this reason, we chose an indirect procedure, aiming to reduce the number of output values $n$ from the neural network. We developed a so-called base function $F({y}_{1}, {y}_{2}, \dots ,{y}_{D})$ for ${y}_{1}, {y}_{2},\dots , {y}_{D} \epsilon {\mathbb{R}}^{C}$ to model the measured pupil data ${d}_{p,meas}(t)$ by varying the model parameters $\{{y}_{i}\}_{i=1}^{D}$. In this way, the temporal pupil response can be reconstructed by knowing the parameters ${y}_{D}$. The primary requirement for the base function is sufficient degrees of freedom $D$, allowing to reconstruct ${d}_{p,out}(t)= F({y}_{1}, {y}_{2}, \dots ,{y}_{D})$ from the empirical pupil data ${d}_{p,meas}(t)$ which is measured in different light spectra conditions $C$. As measured sample set, we had $\{{d}_{p,meas}({t}_{i})\}_{i=1}^{t=300}$ for ${d}_{p,meas}({t}_{i}) \epsilon {\mathbb{R}}^{S\times C}$ available. $S$ denotes the number of subjects in each of the seven stimuli conditions $C$ with the spectra types 420 nm, 530 nm, 610 nm, 660 nm, $\sim$ 2000 K, $\sim$ 5000 K and $\sim$ 10,000 K from the intra- and intersubject experiments. For modelling, the median of the subjects $\{\tilde{d}_{p,meas}(t_{i})\}_{i=1}^{t=300}$ with $\tilde{d}_{p,meas}({t}_{i}) \epsilon {\mathbb{R}}^{C}$ was used. Therefore, the number of subjects $S$ or the performed repetitions in the pupil measurements had no direct effect when training the model. The data sets $\{\tilde{d}_{p,meas}(t_{i})\}_{i=1}^{t=300}$ were used to model each pupil response with the base the function $F({y}_{1}, {y}_{2}, \dots ,{y}_{D})$. As a result, by knowing the model parameters ${\{{y}_{i}\}}_{i=1}^{D}$ for a corresponding stimulus spectrum condition $C$, the temporal pupil diameter ${{\tilde{d}}}_{p,out}(t)$ can be reconstructed with the base function $F({y}_{1}, {y}_{2}, \dots ,{y}_{D})$. The idea is that each temporal median pupil data set $\{\tilde{d}_{p,meas}(t_{i})\}_{i=1}^{t=300}$ from the light conditions $C$ receives its own model parameters $\{{y}_{i}\}_{i=1}^{D}$ with ${y}_{i} \epsilon {\mathbb{R}}^{C}$.

With such an approach, it is no longer necessary to find a direct relationship between associated stimulus quantities $\{{x}_{i}\}_{i=1}^{N}$ and pupil data per time unit $\tilde{d}_{p,meas}(t)$. The indirect approach predicts the model parameters $\{{y}_{i}\}_{i=1}^{D}$ from the respective stimulus quantities $\{{x}_{i}\}_{i=1}^{N}$ using a neural network to insert them into the base function $F({y}_{1}, {y}_{2}, \dots ,{y}_{D})$. Thus, the number of output parameters of the neural network is defined by the degrees of freedom $D$ of the base function $F$. However, the degree of freedom $D$ from the base function $F$ must be sufficient enough to model the measured wave- and time-dependent pupil responses $\tilde{d}_{p,meas}(t)$ (Fig. 1A–C).

### Wavelength-dependent pupil adaptation in the collected train data

**Figure 2:**
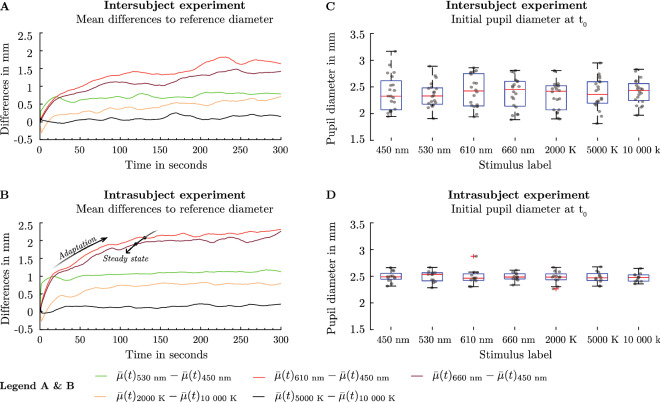
Adaptation behaviour of the pupil in our training data (**A**) Averaged temporal adaptation behaviour of the pupil as a function of different spectra from an intersubject experiment with polychromatic (n: 20, Age: 19–25, Mean age: 21.95 $\pm$ 1.73 y) and chromatic spectra (n: 20, Age: 19–25, Mean age: 22.2 $\pm$ 1.77 y). Subtracting the average pupil diameter from the reference pupil diameter at 450 nm shows that the pupil diameter's equilibrium state is reached at different times. (**B**) Averaged temporal adaptation of the pupil light response from an intrasubject experiment (n: 1, repetitions: 12, Age: 33) with polychromatic and chromatic spectra. (**C**) The initial pupil diameters ${\mathrm{t}}_{0}$ from the intersubject experiment are not significantly affected by the type of the used spectrum $\mathrm{F}(6, 66)=0.85, p=0.537> .05$. (**D**) The measured pupil diameters ${\mathrm{t}}_{0}$ in the intrasubject experiment are not significantly affected by the type of the spectrum $\mathrm{F}(6, 66)= 6.23\cdot {10}^{-2}, p=0.999> .05$.

The pupil's wavelength-dependent adaptation behaviour is essential for a time-dependent model and must be covered in the train data $\{\tilde{d}_{p,meas}(t_{i})\}_{i=1}^{t=300}$. Therefore, we analysed whether the wavelength-dependent temporal behaviour of the afferent pupil path is catched in our data. Using the mean of the pupil diameter $\bar{\mu}(t)_{450 nm}$ as a reference and subtracting it from the other mean values $\bar{\mu}(t)_{530 nm}$, $\bar{\mu}(t)_{610 nm}$, $\bar{\mu}(t)_{660 nm}$, the adaptation behaviour can be related to each other (Fig. 2A, B).

In the intersubject experiment, the comparison of the mean differences showed that the equilibrium state for the spectra 610 nm and 660 nm is reached at 90 s. It takes about 20 s for the 530 nm spectrum (Fig. 2A). The intrasubject experiment showed a more characteristic spectral adaptation behaviour (Fig. 2B). At 610 and 660 nm, the equilibrium status is reached at about 120 s and 530 nm after approximately 10 s. To assess the adaptation response from polychromatic spectra, we used the mean pupil diameter $\bar{\mu}(t)_{10,000 K}$ as a reference. In the intersubject experiment at $\sim$ 2000 K, the adaptation process is completed after 30 s. In the trial with the individual subject, the steady state is reached after 60 s with the $\sim$ 2000 K stimulus. At $\sim$ 5000 K, there is no clear chromatic adaptation either in the individual or in the multiple subject examination because 5700 K was used as pre-stimulus. Thus, the adaptation mechanism is covered in the data and can be considered in the proposed model.

The consequence of the measured time- and wavelength-dependent pupil light response $\{d_{p,meas}(t_{i})\}_{i=1}^{t=300}$ is that it needs to be categorized into a phasic and tonic section, each with the different discussed characteristics. These sections were used to break down the base function $F$ into two “child”-functions before fusing them with into a combined model ${d}_{pM}$(t). The phasic pupil light response represents the constriction of the pupil after a specific latency $\tau$ time from the starting point ${d}_{p,meas}({t}_{1}, \lambda )$ to the peak pupil diameter ${d}_{p,meas}({t}_{\mathrm{Peak}}, \lambda )$ until the beginning of dilatation with ${t}_{1}\le t\le {t}_{d, start}$ (Fig. 1C). In our data, this process takes place approximately in the first two seconds (${t}_{d, start} \approx 2 s$). In the tonic section ${t}_{d,start}\le t\le {t}_{eq}$, the pupil adapts to the stimulus itself under a sustained light stimulus until a state of equilibrium ${t}_{eq}$ is reached. The velocity and gradient of adaptation up to the equilibrium state vary significantly with the spectral distribution $x(\lambda )$. This tonic time ${t}_{eq}$ is defined in our data with 300 s since we measured the pupil diameter in this time window.

### Using the initial pupil diameter to reconstruct the temporal pupil light response

When predicting or reconstructing the pupil response in time, the initial pupil diameter ${d}_{p,meas}({t}_{1}, x(\lambda ))$ is necessary as a starting point. The initial point should preferably be independent of the spectrum, meaning ${d}_{p,meas}({t}_{1}, x(\lambda ))\approx {d}_{p,meas}({t}_{1}, L)$ to facilitate the prediction of the starting position. This would allow the prediction of this pupil diameter ${d}_{p,meas}({t}_{1}, L)$ with a classical L- and M-cone based pupil model. For this purpose, we statistically checked in our data whether the initial pupil diameter is significantly affected by the spectrum $x(\lambda )$ (Fig. 2C, D). According to graphical inspection with a quantile–quantile-plot, normal distributed data can be assumed in both inter- and intrasubject experiments. The Mauchly test revealed for the intersubject examination that the assumption of sphericity had been met $p=0.6> .05$. Therefore, a correction of degree is not needed. According to repeated measure ANOVA, there is no significant difference $F(6, 66)=0.85, p=0.537> .05$ of the initial pupil diameter between the used spectra for the multiple subject trial (Fig. 2C). Within the data from the individual subject, the Mauchly test showed that the assumption of sphericity had been met $p=0.41> .05$ (Fig. 2D). The results from the repeated measure ANOVA showed that the initial pupil diameter is not affected by the type of the spectrum $F(6, 66)= 6.23\cdot {10}^{-2}, p=0.999> .05$. Due to the latency of the pupil and the usage of a constant anchor spectrum, the initial pupil diameter always results from the pre-stimulus at 5700 K. The randomized conduction of the experiments did not significantly affect the initial pupil diameter and we can assume in the following ${d}_{p,meas}({t}_{1}, x(\lambda ))\approx {d}_{p,meas}({t}_{1}, L)$. A wavelength dependence of the initial pupil diameter would have indicated that the anchor pre-stimulus was not presented long enough to adapt the pupil back to its baseline.

### Developing the base functions to model the phasic and tonic pupil light response

There are different time-variant function proposals for the phasic pupil light reflex from the research areas of biomechanics and control engineering. The pupil response is assumed as a time-dependent control loop or mechanical feedback system. With such functions, the phasic pupil course can be reconstructed with corresponding characteristics of the constriction velocity and constriction peak. Unlike the classical L- and M-cone based pupil models, the time-variant function proposals have not been developed with comprehensive empirical data. A valid prediction of the absolute pupil diameter as a function of any intensity magnitude or light spectrum $x(\lambda )$ is not possible without extensive modification.

The function proposals to describe the pupil light reflex as a control system is a so-called black-box approach, which does not provide information about the internal mechanisms of the pupil behaviour^64^. In 1957, Stark et al.^65^ described the pupil light reflex as a servomechanical control system with a delayed linear differential equation of third order. Subsequent work has extended the control loop^66,67^ by using other non-linear differential equations, to create a generalized description of the phasic pupil response^68–72^. Although the proposed control systems describe the behaviour of the phasic pupil light reflex systematically, the transfer functions are not intended to convert them into a closed equation ^73^. In their present proposed form, the functions cannot be used to calculate the pupil diameter as a function of an intensity quantity or spectrum $x(\lambda )$. Furthermore, they do not provide insight into the actual physiological processes of iris muscle activity caused by the parasympathetic and sympathetic nervous system^73^.

Biomechanical approaches break down the pupil light reflex dependencies into individual components, creating functions of the physiological subprocesses for an overall function. In the work of Longtin and Milton^74^, it is discussed that a biomechanical pupil function should include the neuronal feedback control mechanism, spontaneous pupil changes from the autonomic nervous system and the regular oscillation of the pupil^75^. Longtin and Milton^74^ modelled the rate of action potentials in the receptors as a function of luminous flux and then built an equation to describe the efferent signal from the Edinger–Westphal nucleus to the pupil’s muscles. The relationship between pupil muscle activity and the resulting pupil area is derived using the Hill function. A generalized retarded non-linear differential equation is proposed to describe the temporal pupil area as a function of luminous flux. The model parameters of the differential equation depend on muscle activity in the iris.

Pamplona et al.^55^ took this approach and determined the missing constants with the available pupil data from Moon and Spencer^44^. As a result, the function of Longtin and Milton was combined with the model of Moon and Spencer to predict the phasic pupil light reflex as a function of luminance. The resulting model did not consider the fact that Moon and Spencer measured the tonic pupil diameter. Furthermore, the integration of the adaptation phase’s spectral dependence is insufficiently possible due to the proposed function’s low degrees of freedom. The consequence would be a derivation and adaptation of the entire equation for each stimulus condition $C$ in the pupil data $\{\tilde{d}_{p,meas}(t_{i})\}_{i=1}^{t=300}$.

Usui and Hirata^64^ have created a biomechanical pupil function based on iris muscle activity. The constrictor and dilatation muscle are mechanically considered as elastic viscous elements. The equation could be adapted to study data and represent the activity of the autonomic nervous system. However, with a total of 19 differential equations, the entire pupil equation is relatively extensive^73^. Even when the equations are combined, the model still consists of three independent second order delayed differential equations ^73^. A simplified time-variant pupil function was developed by Fan and Yao^73^ with a single delayed differential equation of second degree (Eq. 1). For this purpose, the two iris muscles were modelled separately as viscoelastic materials. The constriction and dilation path were considered separately with the time-dependent muscle forces ${\dot{f}}_{p}(t)$ and ${f}_{s}(t)$.

1 $${d}_{\mathrm{Phasic}}\left(t,{L}_{0d}, {l}_{0c}, {K}_{c}, {K}_{d}, D, {\dot{f}}_{p}, {f}_{s}, {P}_{0} \right)= \frac{{d}^{2}r}{d{t}^{2}}= -{K}_{c}{\left({l}_{0c}-r\right)}^{2}+{K}_{d}{\left({L}_{0d}-r\right)}^{2}-D\frac{dr}{dt}-{\dot{f}}_{p}\left(t\right)+ {f}_{s}\left(t\right)+{P}_{0}$$

${K}_{c}$ and ${K}_{d}$ are the elasticity constants of the constriction and dilatation muscle in the iris. ${L}_{0d}$ and ${l}_{0c}$ define the length of the iris muscles, D the viscosity constant and ${P}_{0}$ the static iris force at resting. The temporal pupil diameter ${d}_{\mathrm{Phasic}}\left(t\right)$ is mainly determined by the time-dependent iris muscle force functions ${\dot{f}}_{p}(t)$ and ${f}_{s}(t)$.

2 $$\dot{f}_{p}(t)=\left\{\begin{array}{lr}
f_{p}+f_{p 0}, & \tau_{p} \leq t \leq \Delta t_{p} \\
f_{p 0}, & t<\tau_{p}, t>\tau_{p}+\Delta t_{p}
\end{array}\right.$$

3 $$f_{s}(t)=\left\{\begin{array}{lr}
f_{s}+f_{s 0}, & \tau_{s} \leq t \leq \Delta t_{s} \\
f_{s 0}, & t<\tau_{s}, t>\tau_{s}+\Delta t_{s}
\end{array}\right.$$

In Eqs. (2) and (3) ${f}_{s0}$, ${f}_{p0}$ are the static iris muscle forces. ${\tau }_{p}$ and ${\tau }_{s}$ define the latency until the respective muscle activity is triggered. The parameters $\Delta {t}_{p}$ and $\Delta {t}_{s}$ represent the duration of the parasympathetic and sympathetic modulation. We decided to use the function of Fan and Yao^73^ to model the phasic pupillary reflex since it combines enough degrees of freedom to fit $\{\tilde{d}_{p,meas}(t_{i})\}_{i=1}^{t=300}$ in any condition of $C$ by changing the model parameters ${X}_{p,Ph}=[{\dot{f}}_{p}(t), {f(t)}_{s}, {P}_{0}, {\tau }_{p}, {\tau }_{s}, \Delta {t}_{p}, {\Delta t}_{s}]$. The values ${X}_{k,Ph}=[{L}_{0d},{l}_{0c},{K}_{d},{K}_{c},D]$ are stimulus independent iris muscle parameters and needs to be calculated once. Coming back to the discussed concept of the neural network, the model parameters ${X}_{p,Ph}\epsilon {\mathbb{R}}^{D1}$ are the first half of values that need to be predicted from the stimulus quantities $\{{x}_{i}\}_{i=1}^{N}$. However, to solve the differential equation numerically, the initial pupil diameter $r(0)={d}_{p,meas}({t}_{1},L)$ must be known. In the previous section, we showed that ${d}_{p,meas}({t}_{1},L)$ is statistically independent of the used spectrum $x(\lambda )$ and resulted from the anchor stimulus. Therefore, we used classical L- and M-cone-based pupil models to predict the starting point ${d}_{p,meas}({t}_{1},L)$. A recent work showed that these models could predict the static equilibrium pupil diameter for white light along the Planckian locus with acceptable prediction errors^25^. We assume that no chromatic stimuli were used as reference light for adaptation, which would also be unusual. The unified model of Watson and Yellot^47^ in Eqs. (4) and (5) was chosen to predict ${d}_{p0}({t}_{1}, L, \alpha ,e)= {d}_{p,meas}({t}_{1}, L)$, because this function was reported as most valuable compared to other L- and M-Cone models^25^.

4 $${d}_{\mathrm{p}0}\left({t}_{1}, L, \alpha ,e\right)=r(0)= {D}_{SDW}\left(L,\alpha ,e \right)+\left(y-{y}_{0}\right)\left[0.02132 -0.009562 \cdot {D}_{SDW}\left(L,\alpha ,e\right)\right]$$

5 $${D}_{SDW}\left(L,\alpha ,e \right)= 7.75- 5.75 \left(\frac{{(L\cdot \alpha \cdot e/846)}^{0.41}}{{(L\cdot \alpha \cdot e/846)}^{0.41} + 2}\right)$$

In the model by Watson and Yellot, the pupil diameter is determined with the parameters $L$ as luminance, $\alpha$ as viewing angle in deg^2^ of the stimulus area and $y$ as the age of a subject. The reference age ${y}_{0}$ is a constant defined by 28.58 years. With such a starting point, the Fan and Yao function is able to fit the temporal phasic pupil diameter $\tilde{d}_{p,meas}(t)$ for ${t}_{1}\le t\le {t}_{d, start}$ well for the different stimulus conditions $C$ but fails to describe the tonic pupil response at ${t}_{d,start}<t\le {t}_{eq}$. The function oscillates for larger time periods, which is not able to describe the wavelength dependent tonic adaptation behaviour (Fig. 2A, B). Therefore, we take a separate function for the tonic pupil response.

We found that a ninth-degree polynomial (Eq. 6) showed appropriate conditions to be considered as a tonic function. It was able to represent any tonic pupil response for each condition $C$ in an automated fitting algorithm. Especially the extreme case where the pupil diameter at short wavelengths is particularly early in equilibrium compared to long wavelengths was covered with this function.

6 $${d}_{\mathrm{Tonic}}\left(t,{a}_{0},{a}_{1},\dots ,{a}_{9}\right)= {a}_{0}+{a}_{1}t+{a}_{2}{t}^{2}+\dots +{a}_{9}{t}^{9}$$

In the following the model parameters ${a}_{0},{a}_{1},\dots ,{a}_{9}$ are defined as ${X}_{p,Ton}\epsilon {\mathbb{R}}^{D2}$. Two masking functions were used to combine the phasic and tonic model into the discussed base function $F$. The masking function ${f}_{\mathrm{Masc}1}(t, q, r)$ is multiplied with the phasic function ${d}_{\mathrm{Phasic}}(t,{X}_{k,Ph}, {X}_{p,Ph})$ and the second masking function ${f}_{\mathrm{Masc}2}(t, q, r)$ with ${d}_{\mathrm{Tonic}}(t,{X}_{p,Ton})$. By combining the two “child”-function, a superposition of both is obtained, which represent a combined light response function ${d}_{pM}(t,q, r,{X}_{k,Ph}, {X}_{p,Ph},{X}_{p,Ton})$ (Eq. 9).

7 $${f}_{\mathrm{Masc}1}(t,q, r)= 1-(0.5+0.5 \cdot \mathrm{tanh}(t-q/r))$$

8 $${f}_{\mathrm{Masc}2}(t,q, r)= 0.5+(0.5 \cdot \mathrm{tanh}(t-q/r))$$

9 $${d}_{pM}(t,\dots )= {d}_{\mathrm{Phasic}}\left(t,{X}_{k,Ph}, {X}_{p,Ph}\right) \cdot {f}_{\mathrm{Masc}1}\left(t,q,r\right)+ {d}_{\mathrm{Tonic}}\left(t,{X}_{p,Ton}\right) \cdot {f}_{\mathrm{Masc}2}\left(t,q,r\right)$$

The parameters of the masking functions $q$ and $r$ determine the position and transition behaviour between the two functions ${d}_{\mathrm{Phasic}}(t,{X}_{k,Ph}, {X}_{p,Ph})$ and ${d}_{\mathrm{Tonic}}(t,{X}_{p,Ton})$. These parameters need to be determined only once and are independent of the pupil data. The resulting base function ${d}_{pM}$ (Eq. 9) can fit the time-dependent pupil data $\{\tilde{d}_{p,meas}(t_{i})\}_{i=1}^{t=300}$ for $\tilde{d}_{p,meas}({t}_{i}) \epsilon {\mathbb{R}}^{C}$ from any experimental measurement condition $C$ and reconstruct it with the respective stimulus-dependent model parameters ${X}_{p}=[{X}_{p,Ph}, {X}_{p,Ton}]$ for ${X}_{p}\epsilon {\mathbb{R}}^{CxD}$. Thus, the temporal pupil light response can be replicated with time-independent model parameters $\{{X}_{p,i}\}_{i=1}^{D}$ in each stimulus condition $C$. The other model parameters $q, r$ and ${X}_{k,Ph}$ can be considered as constants when the function is fitted to $\{\tilde{d}_{p,meas}(t_{i})\}_{i=1}^{t=300}$ in the different stimulus conditions $C$. The combined model (Eq. 9) with the tonic (Eq. 6) and phasic (Eq. 1) function were implemented in MathWorks MATLAB, which is available as an open-source project.

### Computing the model parameters of the phasic and tonic pupil functions

The base function ${d}_{pM}(t,q, r,{X}_{k,Ph}, {X}_{p})$ was used to fit the measured pupil response data $\{\tilde{d}_{p,meas}(t_{i})\}_{i=1}^{t=300}$ in each stimulus conditions $C$ with the spectra 420 nm, 530 nm, 610 nm, 660 nm, $\sim$ 2000 K, $\sim$ 5000 K and $\sim$ 10,000 K. This procedure was performed for both the inter- and intrasubject experiment. The results for the intrasubject experiment are reported in the Supplementary Information. We varied the model parameters ${X}_{p}$ and solved the differential equation numerically by using an ode45 solver, to fit the pupil data. The stimulus independent parameters $q, r$ and ${X}_{k,Ph}$ were determined only once and kept constant for all light conditions to reduce the number of wavelength-dependent parameters. As stated, we calculated the initial pupil diameter ${d}_{\mathrm{p}0}({t}_{1}, L, \alpha ,e)$ with the Watson and Yellot model, using it as a solving condition for the numerical solution of the differential equation. Due to the delayed pupil light response, the anchor spectrum caused the initial pupil diameter. Therefore, the luminance of the anchor spectrum (199.45 cd/m^2^) was set into the Watson and Yellot model. As age parameter, we took the mean value of our sample from the polychromatic (n: 20, Age: 19–25, Mean age: 21.95 $\pm$ 1.73 y) and chromatic (n: 20, Age: 19–25, Mean age: 22.2 $\pm$ 1.77 y) experiment with 22.1 years, resulting in a predicted pupil diameter of 2.79 mm.

However, the measured average initial pupil diameter across all subjects and conditions was 2.38 mm in the dataset. Therefore, an offset correction of 0.41 mm was performed for matching the prediction. The prediction difference is partly due to the fact that our spectrum was generated with a multi-channel LED light whose spectrum differs from the thermal radiators used to develop the Watson and Yellot model. Such an approach was used in a recent publication to adapt classical L- and M-cone based models to pupil data caused by chromatic and polychromatic LED-spectra^25^. The offset corrected prediction of the Watson and Yellot model was used as $r(0)$ in Eq. (1).

We programmed a graphical user interface in MathWorks MATLAB to fit the differential equation to the median of the measured pupil data $\{\tilde{d}_{p,meas}(t_{i})\}_{i=1}^{t=300}$. The software made it possible to change the model parameters ${X}_{p}$ and visualize the solution of the differential equation ${d}_{pM}(t,q, r,{X}_{k,Ph}, {X}_{p})$ (Supplementary Fig. S1) for each lighting condition. We have stored the measured pupil raw data with calculated model parameters (Table 1) for each condition in the available software (see Supplementary Information). The parameters of the masking functions $q$ = 1.1359 and $r$ = 0.3517 were determined manually with the programmed graphical user interface (Supplementary Fig. S1). During the adjustment, we ensured a smooth transition between the phasic and tonic functions in all lighting conditions.

**Table 1:**
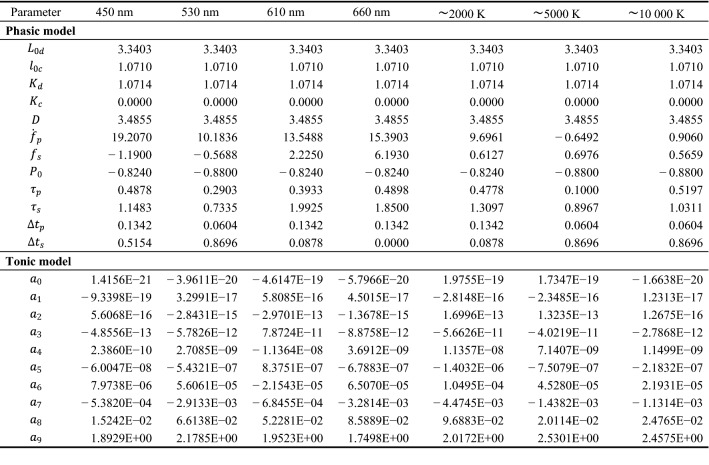
Modell parameters of the phasic and tonic pupil model for each lighting condition in the intersubject study. The values were obtained using a custom programmed user interface in MathWorks MATLAB. The phasic model represents the pupil light response up to two seconds. The data of the remaining pupillary light response are mapped with the tonic model. The median of the sample was used as the target. The sample consisted of 20 subjects (Age: 19–25, Mean age: 21.95 $\pm$ 1.73 y) in the polychromatic investigation and 20 subjects (Age: 19–25, Mean age: 22.2 $\pm$ 1.77 y) in the chromatic investigation. The predicted pupil diameter from the offset corrected Watson and Yellot model was used as $r\left(0\right)$ to solve the differential equation. The mean value of the two age groups with 22.1 y, the luminance of the anchor spectrum with 199.45 cd/m^2^ and the size of the adaptation surface with 53.1° were used as parameters in the Watson and Yellot model. An offset of 0.41 mm was subtracted to adjust the model to our data.

As a result of this approach, 17 dependent and seven constant values represent the temporal pupil light response for each light condition. Using the base function ${d}_{pM}(t,q, r,{X}_{k,Ph}, {X}_{p})$, we have reduced the feature set from 300 pupil diameter values (1 s resolution) to 17 model parameters ${X}_{p}$. Thus, by combining a neural network with the base function, the time and wavelength-dependent pupil diameter can be reconstructed by predicting ${X}_{p}$ from the stimulus quantities $\{{x}_{i}\}_{i=1}^{N}$.

### Linking stimulus quantities with model parameters through a neural network

The knowledge of model parameters ${X}_{p}$ alone is not advantageous because the connection to the stimulus characteristics $\{{x}_{i}\}_{i=1}^{N}$ in each condition $C$ is missing. Therefore, we used the calculated stimulus dependent parameters ${X}_{p}$ of the base function to train a neural network with photometric, colourimetric or receptor signals as input parameters (Table 1). We aimed to establish a link between the model parameters ${X}_{p}$ of the base function and stimulus quantities ${\{{x}_{i}\}}_{i=1}^{N}$. Ideally, this would ensure that after the input of stimulus values from a stimulus condition such as luminance and CIExy-2° chromaticity points, the respective model parameters ${X}_{p}$ from Table 1 could be predicted through the neural network. The reconstruction of the temporal pupil light reflex ${d}_{p,out}({t}_{1}, {t}_{2},\dots , {t}_{n})$ would be possible by solving the base function ${d}_{pM}(t,q, r,{X}_{k,Ph}, {X}_{p})$ with the predicted values ${X}_{p}$ from the neural network. At first, we need to determine which combination stimulus features make sense as input parameters to the neural network. We trained three variants of feedforward neural networks, each with different input combinations. From the measured stimulus spectra $x(\lambda )$, we calculated the photometric, colorimetric and receptor-based quantities and used the mean stimulus values (Table 2) for training. We used the input parameters luminance and the CIExy-2° chromaticity points for the neural network's first variant $\{{x}_{v1,i}\}_{i=1}^{N=3}$. Variant two $\{{x}_{v2,i}\}_{i=1}^{N=4}$ was trained with the L-, M-, S-cones and the melanopsin signals. The luminance, CIExy-2° chromaticity points and the melanopsin signal was used in the third variant $\{{x}_{v3,i}\}_{i=1}^{N=4}$. The train data sets were normalized with the unity-based normalization ${X}_{i}={(X}_{i}-{X}_{\mathrm{Min}})/({X}_{\mathrm{Max}}- {X}_{\mathrm{Min}})$ before the training was conducted.

**Table 2:** Metrics that were used as features for the neural network. The features were calculated from the repeated measured spectra in the pupil examinations^25^. The values are given with standard deviation in the table, but for training the neural network, the mean values were used. On each study day, stimuli were measured twenty times with a Konica Minolta CS-2000 spectroradiometer. S-cone, M-cone, L-cone and ipRGC excitation were calculated with the 10-deg cone fundamentals and the melanopic action spectra reported in CIE S 026/E:2018. The cone and ipRGCs excitation values are specified as $\alpha$-opic radiance in W/m^2^sr.

Stimulus label	Luminance in cd/m^2^	CIEx 1931 2°	CIEy 1931 2°	L-cone	M-cone	S-cone	Melanopsin	CCT In Kelvin
${\lambda }_{\mathrm{Peak}}$ 450 nm	99.73 $\pm$ 0.4	0.1581 $\pm$ 1.57 $\cdot$ 10^–5^	0.02006 $\pm$ 5.26 $\cdot$ 10^–5^	0.29130 $\pm$ 8.7 $\cdot$ 10^–4^	0.48338 $\pm$ 1.2 $\cdot$ 10^–3^	3.24582 $\pm$ 6.2 $\cdot$ 10^–3^	1.81725 $\pm$ 3.9 $\cdot$ 10^–3^	–
${\lambda }_{\mathrm{Peak}}$ 530 nm	100.12 $\pm$ 0.2	0.18661 $\pm$ 1.17 $\cdot$ 10^–4^	0.73928 $\pm$ 1.07 $\cdot$ 10^–4^	0.14091 $\pm$ 3.3 $\cdot$ 10^–4^	0.16724 $\pm$ 3.8 $\cdot$ 10^–4^	0.00396 $\pm$ 1.4 $\cdot$ 10^–5^	0.10547 $\pm$ 2.3 $\cdot$ 10^–4^	–
${\lambda }_{\mathrm{Peak}}$ 610 nm	100.16 $\pm$ 0.2	0.67903 $\pm$ 1.08 $\cdot$ 10^–4^	0.32039 $\pm$ 4.40 $\cdot$ 10^–5^	0.19684 $\pm$ 4.6 $\cdot$ 10^–4^	0.04708 $\pm$ 1.0 $\cdot$ 10^–4^	0.00009 $\pm$ 3.5 $\cdot$ 10^–5^	0.00075 $\pm$ 2.8 $\cdot$ 10^–5^	–
${\lambda }_{\mathrm{Peak}}$ 660 nm	99.97 $\pm$ 0.2	0.71701 $\pm$ 3.40 $\cdot$ 10^–4^	0.27995 $\pm$ 1.07 $\cdot$ 10^–4^	0.20227 $\pm$ 4.4 $\cdot$ 10^–4^	0.02363 $\pm$ 7.1 $\cdot$ 10^–5^	0.00084 $\pm$ 1.1 $\cdot$ 10^–4^	0.00114 $\pm$ 1.3 $\cdot$ 10^–4^	–
Polychromatic $\sim$ 2000 K	100.17 $\pm$ 0.3	0.53305 $\pm$ 4.90 $\cdot$ 10^–5^	0.42288 $\pm$ 3.82 $\cdot$ 10^–5^	0.17090 $\pm$ 4.1 $\cdot$ 10^–4^	0.10258 $\pm$ 2.6 $\cdot$ 10^–4^	0.00637 $\pm$ 2.1 $\cdot$ 10^–5^	0.04561 $\pm$ 1.2 $\cdot$ 10^–4^	2007 $\pm$ 1
Polychromatic $\sim$ 5000 K	100.10 $\pm$ 0.4	0.34538 $\pm$ 1.02 $\cdot$ 10^–4^	0.34976 $\pm$ 1.05 $\cdot$ 10^–4^	0.16452 $\pm$ 3.5 $\cdot$ 10^–4^	0.13957 $\pm$ 2.8 $\cdot$ 10^–4^	0.06364 $\pm$ 1.2 $\cdot$ 10^–4^	0.12247 $\pm$ 2.4 $\cdot$ 10^–4^	4983 $\pm$ 3
Polychromatic $\sim$ 10,000 K	99.83 $\pm$ 0.2	0.28549 $\pm$ 1.02 $\cdot$ 10^−4^	0.27690 $\pm$ 1.40 $\cdot$ 10^–4^	0.16520 $\pm$ 3.7 $\cdot$ 10^–4^	0.15241 $\pm$ 3.3 $\cdot$ 10^–4^	0.11978 $\pm$ 2.3 $\cdot$ 10^–4^	0.16680 $\pm$ 3.3 $\cdot$ 10^–4^	10,138 $\pm$ 22

The neural networks were trained and implemented using PyTorch 1.5 with PyTorch Lightning^76^ in Python 3. We trained the model by minimizing the mean squared error $MSE= 1/N{\sum }_{i=1}^{n}{({y}_{i}- {y}_{0i})}^{2}$ between the output of the neural network ${y}_{i}$ and the target model parameters ${y}_{0i}$ (Table 1, Supplementary Table S1). The weightings were optimized using a Adam optimizer^77^, with a learning rate of 0.001 and a batch size of 7. We used three fully connected layers (40, 380, 80) with a rectified linear unit (ReLu) activation function. The number of neurons of the input layer corresponded to the number of input parameters $N$ (Variant 1: 3, Variant 2: 4, Variant 3: 4) and the number of neurons of the output layer was 17. Three fully connected hidden layers were used with 40, 380 and 80 neurons, respectively. The neural networks were trained 4000 epochs (Supplementary Fig. S2) by using the calculated model parameters ${X}_{p}\epsilon {\mathbb{R}}^{C}$ with $C$ as stimulus conditions. For each variant, two neural network versions were trained. One based on the intersubject parameters (Table 1) and the second with the intrasubject parameters (SupplementaryTable S1). The training process over the epochs is reported in Supplementary Fig. S2.

## Results

### The deep learning-driven pupil model approach

**Figure 3:**
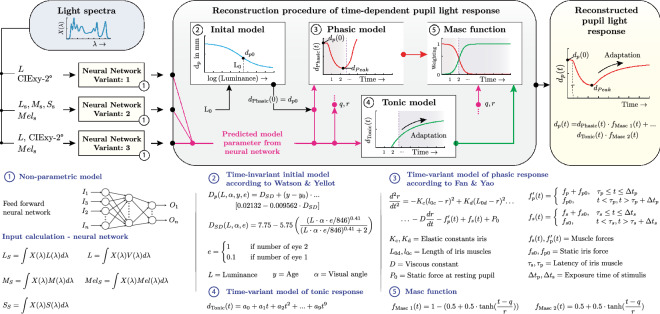
Structure of the proposed overall model for reconstructing the temporal pupil response based on neural networks. **Step 1:** The derived stimulus characteristics are entered into the neuronal network and the model parameters of the phasic and tonic model are predicted. **Step 2:** The initial pupil diameter is determined with the Watson and Yellot model from the luminance of the anchor spectrum. **Step 3:** The initial pupil diameter and the predicted model parameters from the neural network are used to determine the numerical solution of the phasic model, which is based on the Fan and Yao differential equation. **Step 4:** The second part of the predicted model parameters from the neural network is used for the tonic model. **Step 5:** The results of the phasic and tonic model are combined with two masking functions to reconstruct the complete pupil light response caused by a respective photometric or receptor-based quantity.

The structure of the overall model proposal to reconstruct the time-dependent pupil response ${{\tilde{d}}}_{p,out}({t}_{1}, {t}_{2},\dots , {t}_{n})$ with a neural network as a data-driven component is summarized in Fig. 3. After the neural networks have been trained (Variant 1 to 3) with the corresponding data sets (Table 1, Supplementary S1, S2), they are able to output the model parameters of the tonic ${X}_{p,Ton}$ and phasic ${X}_{p,Ph}$ functions from photometric or receptor-based quantities ${x}_{v1},{x}_{v2}$ and ${x}_{v3}$ (Fig. 3: Step 1).

We achieved a robust neural network-driven prediction accuracy of the model parameters with the lighting metric features. Among the neural networks with the intersubject model parameters (Table 1), the first variant ${x}_{v1}$ with CIExy-2° chromaticity points and luminance as input achieved the lowest loss after 1139 epochs (MSE: $2.11 \cdot {10}^{-15}$, MAE: $3.19\cdot {10}^{-8}\ \mathrm{SD}\pm 3.31\cdot {10}^{-8}$). Variant two ${x}_{v2}$ with the receptor and melanopsin signals as input achieved a mean squared error (MSE) of $1.07 \cdot {10}^{-14}$ and mean absolute error (MAE) of $7.02 \cdot {10}^{-8}\ \mathrm{SD}\pm 7.63\cdot {10}^{-8}$ after 3844 epochs. The third variant ${x}_{v3}$ with the luminance, CIExy-2° chromaticity points and melanopsin signal as input parameter reached an MSE of $2.63 \cdot {10}^{-15}$ and MAE of $3.56 \cdot {10}^{-8}\ \mathrm{SD}\pm 3.69\cdot {10}^{-8}$ after 1056 epochs. In the neuronal networks that were trained with the intrasubject model parameters (Supplementary Table S1), variant 1 (MSE: $3.1 \cdot {10}^{-15}$, MAE: $3.99\cdot {10}^{-8}\ \mathrm{SD}\pm 3.84\cdot {10}^{-8}$, epoch: 2207) and variant 3 (MSE: $2.28 \cdot {10}^{-15}$, MAE: $3.20\cdot {10}^{-8}\ \mathrm{SD}\pm 3.55\cdot {10}^{-8}$, epoch: 3163) reached a higher accuracy compared to variant 2 (MSE: $2.87 \cdot {10}^{-14}$, MAE: $1.08\cdot {10}^{-7}\ \mathrm{SD}\pm 1.31\cdot {10}^{-7}$, epoch: 2907). Thus, we were able to use the neural networks’ predicted model parameters ${X}_{p}$ in the base function (Eq. 9), calculated from photometric and colorimetric or receptor-based quantities (Fig. 3: Step 1).

The next step in the model is to determine the initial pupil diameter ${d}_{p0}({t}_{1}, L, \alpha ,e)$ with the Watson and Yellot model (Fig. 3: Step 2). It is inserted as an initial state ${d}_{p}(0)$ together with the predicted model parameters of the neural network (Fig. 1: Step 1) into the second order differential equation ${d}_{\mathrm{Phasic}}(t,{X}_{k,Ph}, {X}_{p,Ph})$ and solved numerically to reconstruct the phasic pupil light response. The second part of the predicted model parameters ${X}_{p,Ton}$ from the neural network is applied to the tonic model ${d}_{\mathrm{Tonic}}(t,{X}_{p,Ton})$ to reconstruct the pupil course from the peak pupil diameter to the equilibrium state (Fig. 3: Step 4). This part is particularly important for mapping the wavelength- and time-dependent adaptation of the pupil control path (Fig. 1, 2). In the last step, the prediction from the phasic and tonic model is combined by the masking functions (Eqs. 7, 8) according to the combined model equation (Eq. 9), to obtain the total reconstructed pupil response up to 300 s. Thus, the entire time course of the pupil light response can be determined by using photometric or receptor-based quantities. In this overall system, the neuronal networks represent the data-driven component. The structure in Fig. 3 is embedded in an algorithm in MathWorks MATLAB and Python, allowing to return the complete temporal pupil response through the respective stimulus quantities.

### Reconstructing the temporal pupil light response with the proposed model approach

**Figure 4:**
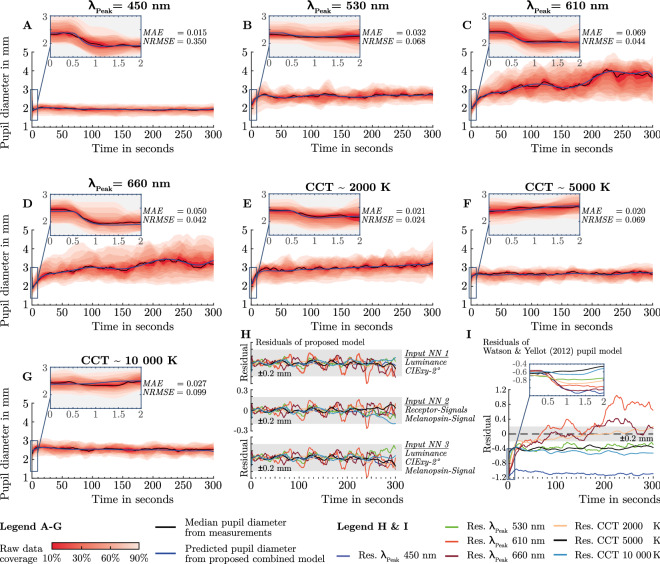
Results of the trained combined model approach with the intersubject dataset. (**A–G**) The measured median pupil diameter is plotted with the percentiles as a shaded area. The predicted reconstructed pupil diameter of our proposed model concept is drawn as a blue line for comparison. The neural network variant one, with the luminance and the CIExy-2° chromaticity coordinates, was used to predict the model parameters. (**H**) Calculated residuals from the measured median pupil diameter for each lighting condition. The residuals were calculated by running the whole model with different neural network variants to predict the model parameters for the phasic and tonic function. (**I**) Calculated residuals from the Watson and Yellot model for each light condition compared to the measured median pupil diameter.

We used the discussed structure of the proposed pupil model approach (Fig. 3) and the trained neural networks to perform a direct comparison between the measured pupil diameter from the intersubject experiments and the predicted reconstructed pupil response. Figure 4 (A–G) shows the measured median pupil diameter and the predicted pupil response (Variant 1) for each lighting condition. The median pupil diameter is plotted with the respective percentile range of the raw data.

The mean absolute error (MAE) between measured and predicted pupil diameter is between 0.015 mm and 0.069 mm for chromatic and polychromatic stimuli. The residuals analysis showed that for each variant of a neural network, the prediction error of the proposed concept is below $\pm$ 0.3 mm (Fig. 4H). At most times, the error is even less than $\pm$ 0.2 mm. Just with the stimulus of ${\lambda }_{\mathrm{Peak}}$ = 610 nm, an eruption of up to -0.3 mm prediction error is observed between 240 and 250 s, which is due to fluctuations of the median diameter (Fig. 4C). The same analysis was performed for the trained combined model with the intrasubject data sets, showing that the error was even smaller than for the intersubject data, due to the lower fluctuation of the median diameter (Supplementary Fig. S3).

As a comparison to our model concept, we calculated the residuals of the classical L- and M-cone based pupil model by Watson and Yellot in relation to the measured median diameter (Fig. 4I). The prediction of the Watson and Yellot model had an absolute prediction error of greater than 0.6 mm for the phasic pupil diameter. For the tonic pupil diameter, the error increases to 1.14 mm due to the time- and wavelength-dependent dependent receptor weighting of the pupil path (Fig. 4I), showing that the inaccuracy of the L- and M-cone based pupil model is not only caused by the lack of melanopsin weighting.

## Discussion

The key idea of this work is to model the temporal pupil light response for different stimuli through a time-variant biomechanical differential equation and predict its model parameters using a deep learning approach. We showed that the concept works well for both chromatic and polychromatic spectra with a mean absolute error of less than 0.1 mm across the 300 s of the pupil's time course. The trained neural networks were able to find a pattern between the light parameter features and the model parameter successfully. All input parameter combination ${x}_{v1}$, ${x}_{v2}$, and ${x}_{v2}$ achieved a loss that would allow the usage in the proposed combined model. Furthermore, the fusion of the combined model with neural networks revealed that with all three light-metric feature combinations, the residuals were in a range of $\pm$ 0.2 mm. Similar results were obtained with the intrasubject dataset, indicating the validity of the proposed pupil modelling concept. Specifically, the first input variant ${x}_{v1}$ could make a simplified application possible^78^ since only the CIExy-2° chromaticity points and the luminance of a stimulus is necessary for determining the base function's model parameters and reconstructing the temporal pupil light response.

Compared to the recently published models by Holladay^42^ and Crawford^43^, Moon and Spencer^44^, De Groot and Gebhard^45^, Stanley and Davies^46^, Watson and Yellot^47^ and Rao et al*.*^48^, we took additionally into account the temporal, spectral receptor weighting of the afferent pupil control path. We can predict the pupil's spectral dependent phasic and tonic time course up to 300 s adaptation time which outperforms previous approaches. Additionally, the combined model is non-parametric, meaning a continuous extension of the prediction space through data basis upgrades is possible without changing the basic structure. Analysation of the residuals from the Watson and Yellot function (Fig. 4I), showed that in pupil modelling the spectral dependence need to be considered together with the time behaviour. The adaptive weighting of the ipRGCs leads to different tonic pupil response patterns depending on the stimulus spectra. Therefore, previous approaches are currently reaching their limits and cannot be extended to solve the issue of pupil modelling.

Note that the neural networks' input values are used to support the pattern recognition between the input features and predicted model parameters of the basis function. At the moment, our input parameters are used for classifying the respective stimulus spectrum without considering external study dependent parameters such as the adaptation field size $\alpha$. For instance, we used the CIExy-2° coordinates although the adaptation field size in our setup corresponded to a visual angle of 53.1°. Suppose the neural network should also manage the pupil's relationship between different adaptation field sizes. In that case, it makes more sense of using a separate parameter $\alpha$ as input to the model in the future. A simultaneous change of the CIExy observer is not needed, because the chromaticity point features are only intended for specifying the stimulus itself without considering the adaptation field size. Thus, each input feature should have its identification task of a stimulus or experimental condition modality. However, it will be interesting to what extent the currently used input parameters behave when using pupil data caused from metamer stimuli, i.e. different spectra with the same chromaticity points. We assume that in such a case, additionally to luminance and CIExy-2° coordinates, the melanopsin signal needs to be integrated as an input (input variant ${x}_{v3}$) for characterizing the stimulus.

Our proposed combined model is currently based on the temporal pupil light of seven different spectra a constant luminance, which is insufficient for a finalized pupil light response model. When focusing on the future perspective of our approach, it is necessary to train the neural networks with an additional amount of temporal pupil data, ensuring continuous development of the stimulus modalities' prediction space. With sufficient training data, it should be possible of reconstructing the temporal pupil light response even for stimulus metrics that are explicitly not present in the training data. However, taking into account the amount of the pupil's control path influencing parameters, the data collection must be prioritized. In our view, the next step is to collect data on the pupil light response to fully model the behaviour with varying luminance and spectral power distributions by using the silent substitution technique^79^. For this purpose, the parameters of the anchor's luminance, anchor's spectrum and exposure time of the main stimulus should not be varied as this leads to additional influencing parameters, impairing the training result of the neuronal network. As the next important step, we consider the modelling of the exposure time, which would require a similar experimental protocol but with different adaptation times of the main stimulus. Due to the non-parametric model approach, the adaptation time could be mapped to the neuronal network as an additional input parameter, if sufficient training data is available. In the same way, other influencing parameters such as the adaptation field size $\alpha$ or cognitive effects could be increasingly incorporated into the combined model to approach a comprehensive pupil behaviour description with new data dependency layers.

A weakness of the proposed model is the integrated polynomial equation for describing the tonic pupil behaviour. The tonic function alone requires ten input parameters, which need to be predicted by the neural network. In principle, this has not led to any disadvantage in reconstructing the temporal pupil light response. However, this approach is not elegant, making an alternate function with a smaller number of parameters preferable. This is an open issue which we need to address in an upcoming work. Furthermore, we currently assume a static reference spectrum (anchor) as an adaptation in our proposed model. If one wants to model the temporal pupil light reflex relating to different anchor spectra, it is not sufficient to change the starting point ${d}_{p}(0)$ of the pupil course with the Watson and Yellot component (Fig. 3: Step 2). Although the Watson and Yellot model determines the starting point ${d}_{p}(0)$ of the pupil's course, a change in the reference spectrum or luminance also means that the entire pupil light response could be different, affecting the tonic ${X}_{p,Ton}$ and phasic ${X}_{p,Ph}$ model parameters. In fact, for modelling the relationship between different adaptation spectra and the pupil light response from a main stimulus, the combined model needs an adaptation input in the neural network additionally. In general, one must consider that a higher number of input parameters in the neural network leads to a more robust prediction for additional dependencies, but simultaneously to a more complex application of the model, because more parameters have to be entered. In future, only the neural network’s input count need to be changed if more dependencies should be modelled since the base function has a sufficient degree of freedom for describing any temporal pupil response.

The research applications in the field of pupillometry are highly interdisciplinary^80–88^ across species^89^, covering the topic of clinical diagnostics^41, 90–95^, cognitive science^96–103^, neuroscience^104^, vision science^105,106^, autonomous nervous system^107–109^ and quantification of the circadian photoentrainment^39,110–113^. A reliable data-driven pupil model that integrates the findings of past years could also be an essential step forward for these research areas. However, individual research groups will not be able to model the pupil behaviour’s cognitive and light-induced dependencies alone, so the focus should be in our view on a non-parametric data-driven approach^114^. Therefore, in future works, we will connect the current combined model with a publicly accessible pupil database, achieving an automated self-maintenance of the neural networks as the database grows. The entire code and neural networks are provided with this manuscript so that this concept could become a door-opener to an overall model of the light- and cognitive induced pupil dependencies.

## Supplementary information

Supplementary Information 1.

## Data Availability

The training data, graphical toolbox and the implemented pupil model with respective neural networks is available at the main authors’ GitHub page: https://github.com/BZandi/DL-PupilModel.
