# Supplementary material for: Deep learning-based pupil model predicts time and spectral dependent light responses
Source: Sci Rep. 2021 Jan 12;11:841. doi: 10.1038/s41598-020-79908-5 (PMC7803766; doi:10.1038/s41598-020-79908-5)
Supplement: Supplementary file 1 — Supplementary Information 1. [file 41598_2020_79908_MOESM1_ESM.pdf]

Deep learning-based pupil model predicts time and spectral dependent light responses

Supplementary materials

Babak Zandi & Tran Quoc Khanh
Technical University of Darmstadt
Department of Electrical Engineering and Information Technology
Laboratory of Lighting Technology, D-64289 Darmstadt in Germany

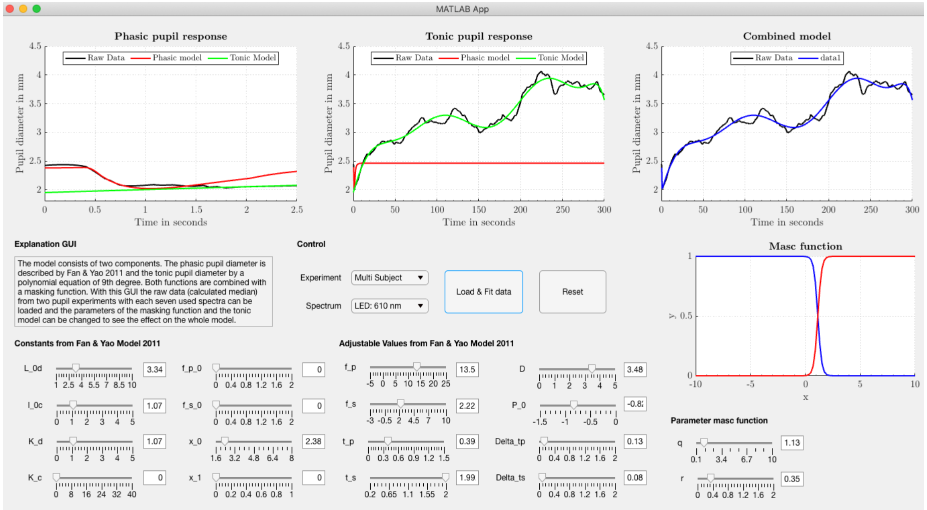

Figure S1 To determine the model parameters of the Fan & Yao phasic function, we programmed a custom graphical user interface in MathWorks MATLAB. The measured median pupil diameter can be loaded, and it is possible to adjust the function by changing the model parameters. The differential equation is directly solved numerically and plotted when moving the sliders. The model parameters used in this manuscript and the median pupil diameter for the intra- and interpersonal experiments are stored as basic data sets. The software is available in GitHub: https://github.com/BZandi/DL-PupilModel

Table S1 Model parameters of the phasic and tonic pupil model for each lighting condition in the intrasubject study. The values were obtained using a custom programmed user interface in MathWorks MATLAB. The phasic model represents the pupil light response up to two seconds. The data of the remaining pupillary light response are mapped with the tonic model. The median of the sample was used as the target. The sample consisted of one subject with 12 repetitions (Age: 33). The predicted pupil diameter from the offset corrected Watson & Yellot model was used as $r(0)$ to solve the differential equation. The age of the subject, the luminance of the anchor spectrum with 199.45 cd/m² and the size of the adaptation surface with 53.1° were used as parameters in the Watson & Yellot model. An offset of 0.25 mm was subtracted to adjust the model to our data.

Parameter	λ_{Peak} 450 nm	λ_{Peak} 530 nm	λ_{Peak} 610 nm	λ_{Peak} 660 nm	CCT 2000 K	CCT 5000 K	CCT 10 000 K
				**Phasic model**			
L_{0d}	3.3403	3.3403	3.3403	3.3403	3.3403	3.3403	3.3403
l_{0c}	1.0710	1.0710	1.0710	1.0710	1.0710	1.0710	1.0710
K_d	1.0714	1.0714	1.0714	1.0714	1.0714	1.0714	1.0714
K_c	0.0000	0.0000	0.0000	0.0000	0.0000	0.0000	0.0000
D	3.4855	3.4855	3.4855	3.4855	3.4855	3.4855	3.4855
$\dot{f}_p$	20.0000	-0.6492	13.9306	15.3903	5.3645	-0.6492	0.5889
f_s	-1.1900	1.5375	2.2250	6.1930	0.6127	0.6976	1.1245
P_0	-0.8240	-0.8800	-0.8240	-0.8240	-0.8240	-0.8800	-0.8800
τ_p	0.4878	0.1000	0.4722	0.4377	0.4018	0.1000	0.1000
τ_s	1.2943	0.7335	1.4561	1.8500	1.3097	0.4306	0.9682
Δt_p	0.1342	0.0604	0.1342	0.1342	0.1342	0.0604	0.0604
Δt_s	0.0878	0.8696	0.0878	0.0878	0.0878	0.8696	0.8696
				**Tonic model**			
a_0	2.7044E-20	7.7429E-20	1.6555E-19	2.2084E-20	2.3869E-19	1.5122E-20	6.3540E-20
a_1	-3.7830E-17	-1.0897E-16	-2.3978E-16	-4.8750E-17	-3.3793E-16	-2.3891E-17	-8.9538E-17
a_2	2.2550E-14	6.4824E-14	1.4801E-13	4.1248E-14	2.0206E-13	1.5656E-14	5.3360E-14
a_3	-7.4815E-12	-2.1250E-11	-5.0729E-11	-1.8156E-11	-6.6397E-11	-5.5593E-12	-1.7510E-11
a_4	1.5105E-09	4.1945E-09	1.0515E-08	4.6196E-09	1.3067E-08	1.1738E-09	3.4465E-09
a_5	-1.9003E-07	-5.1091E-07	-1.3426E-06	-7.0132E-07	-1.5736E-06	-1.5284E-07	-4.1417E-07
a_6	1.4593E-05	3.7722E-05	1.0297E-04	6.2720E-05	1.1366E-04	1.2383E-05	2.9372E-05
a_7	-6.3581E-04	-1.5827E-03	-4.4875E-03	-3.1717E-03	-4.6465E-03	-6.1209E-04	-1.1051E-03
a_8	1.2399E-02	3.3025E-02	1.1081E-01	9.1798E-02	9.9727E-02	1.6490E-02	1.6234E-02
a_9	1.9227E+00	2.7055E+00	2.0117E+00	1.9910E+00	2.1998E+00	2.5868E+00	2.6331E+00

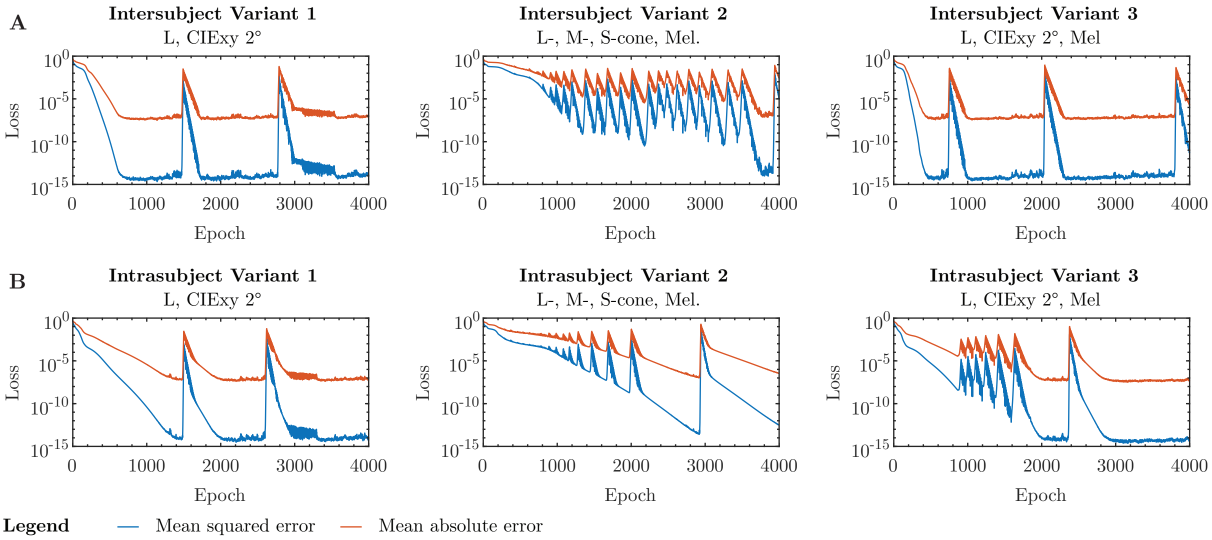

Figure S2 Plotted Loss of the neural networks during training for each light condition. Both the men squared error and the mean absolute error is plotted. A: Calculated loss for each epoch for the neuronal networks that were trained with the interpersonal dataset. B: Calculated Loss for each epoch for the neuronal networks that were trained with the intrapersonal dataset.

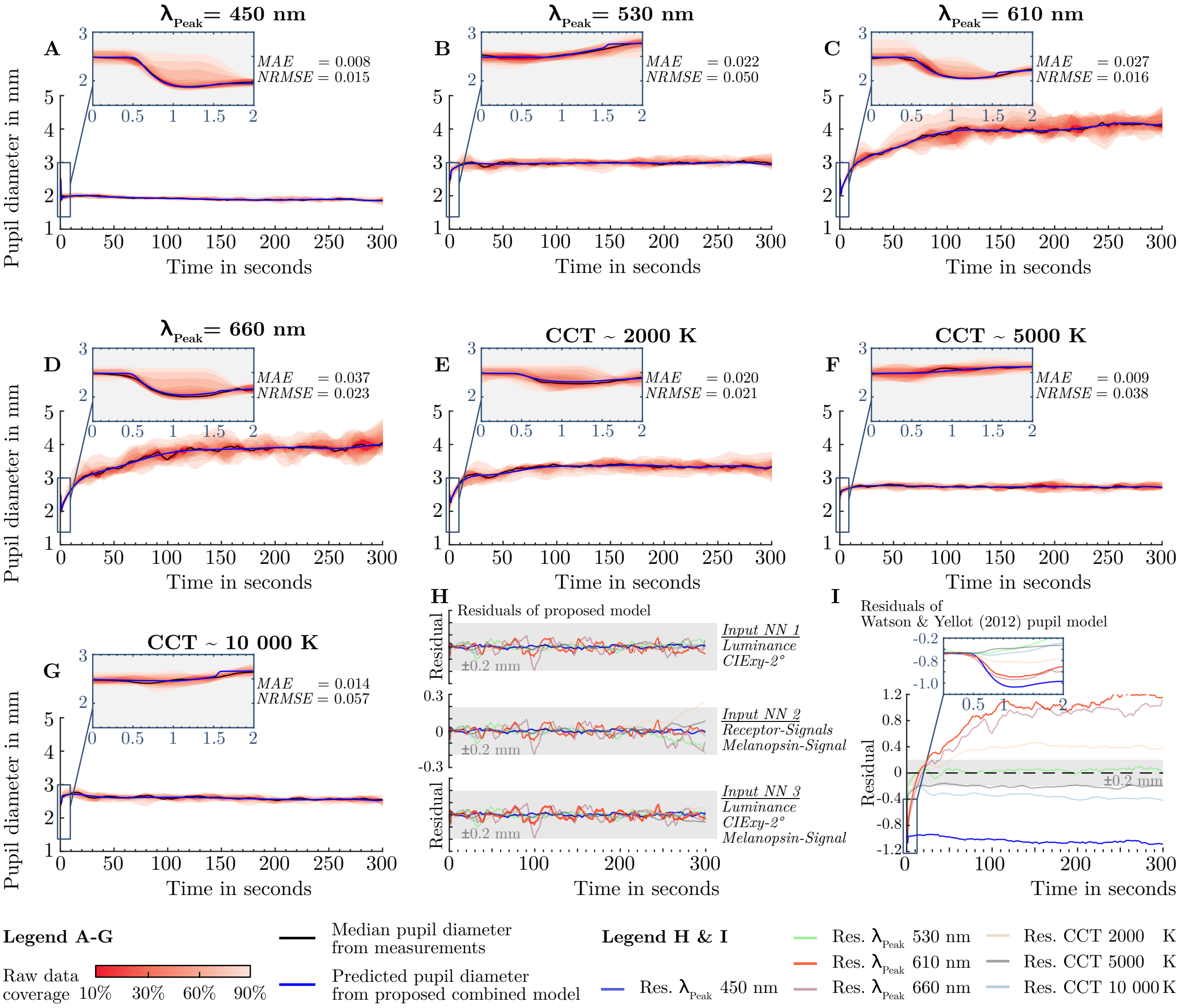

Figure S3 Results of the trained combined model approach with the intrasubject dataset. **A-G:** The measured median pupil diameter is plotted with the percentiles as a shaded area. For comparison, the predicted reconstructed pupil diameter of our proposed model concept is drawn as a blue line. The neural network variant one with the luminance and the CIExy-2° chromaticity coordinates was used to predict the model parameters. **H:** Calculated residuals from the measured median pupil diameter for each lighting condition. The residuals were calculated by running the whole model with different neural network variants to predict the model parameters for the phasic and tonic function. **I:** Calculated residuals from the Watson & Yellot model for each light condition compared to the measured median pupil diameter.

Table S 2 Measured absolute spectra in W/m^{2}sr of the main and anchor stimuli used in the pupil experiment conducted in the work "Prediction accuracy of L- and M-cone based human pupil light models" published in Scientific Reports (DOI: https://doi.org/10.1038/s41598-020-67593-3). Spectra were measured with a Konica Minolta CS2000 spectroradiometer. Wavelength step size was reduced to 5 nm.

Wavelength	Anchor 5500 K	2000 K	5000 K	10 000 K	450 nm	530 nm	610 nm	660 nm
380	2.69E-06	2.47E-06	2.95E-06	3.79E-06	6.62E-05	7.51E-07	7.18E-06	7.51E-05
385	1.92E-06	0.00E+00	0.00E+00	0.00E+00	1.65E-04	2.23E-06	1.34E-06	5.41E-05
390	0.00E+00	0.00E+00	0.00E+00	0.00E+00	6.78E-05	5.28E-07	0.00E+00	2.13E-05
395	0.00E+00	0.00E+00	0.00E+00	5.38E-07	1.49E-05	1.42E-06	0.00E+00	1.24E-06
400	0.00E+00	0.00E+00	4.04E-06	1.43E-05	2.46E-05	0.00E+00	1.37E-06	3.52E-05
405	7.45E-06	9.20E-07	1.84E-05	8.26E-05	3.17E-04	3.67E-07	2.52E-07	2.66E-05
410	2.92E-05	3.62E-06	7.17E-05	3.17E-04	1.20E-03	1.19E-06	0.00E+00	1.09E-05
415	1.00E-04	6.69E-06	2.11E-04	9.70E-04	3.85E-03	0.00E+00	6.96E-06	1.04E-05
420	2.62E-04	1.39E-05	4.60E-04	2.11E-03	9.95E-03	0.00E+00	0.00E+00	3.48E-05
425	5.88E-04	2.10E-05	5.94E-04	2.50E-03	2.23E-02	7.14E-07	0.00E+00	1.05E-05
430	1.14E-03	2.76E-05	5.41E-04	1.75E-03	4.20E-02	2.71E-06	0.00E+00	6.40E-06
435	2.01E-03	4.24E-05	6.88E-04	1.67E-03	7.27E-02	2.36E-06	0.00E+00	2.76E-05
440	3.55E-03	7.22E-05	1.12E-03	2.35E-03	1.18E-01	1.04E-06	0.00E+00	1.01E-05
445	5.47E-03	1.19E-04	1.73E-03	3.39E-03	1.54E-01	1.24E-06	1.57E-07	3.10E-06
450	5.13E-03	1.52E-04	1.85E-03	3.35E-03	1.30E-01	2.96E-06	0.00E+00	9.04E-06
455	3.09E-03	1.50E-04	1.46E-03	2.27E-03	7.58E-02	2.74E-06	0.00E+00	1.85E-07
460	2.28E-03	1.20E-04	1.22E-03	1.80E-03	4.43E-02	3.83E-06	0.00E+00	2.11E-06
465	1.79E-03	1.00E-04	1.24E-03	1.84E-03	2.66E-02	7.34E-06	4.40E-07	0.00E+00
470	1.23E-03	9.49E-05	1.42E-03	2.13E-03	1.44E-02	1.33E-05	0.00E+00	1.06E-05
475	1.07E-03	9.50E-05	1.54E-03	2.38E-03	8.24E-03	2.76E-05	0.00E+00	0.00E+00
480	1.12E-03	1.11E-04	1.40E-03	2.12E-03	4.99E-03	5.81E-05	1.81E-06	0.00E+00
485	1.22E-03	1.63E-04	1.16E-03	1.61E-03	2.92E-03	1.18E-04	0.00E+00	1.44E-05
490	1.47E-03	2.58E-04	1.08E-03	1.35E-03	1.73E-03	2.34E-04	0.00E+00	1.36E-05
495	1.79E-03	4.16E-04	1.20E-03	1.38E-03	1.07E-03	4.55E-04	0.00E+00	3.75E-06
500	2.10E-03	6.40E-04	1.44E-03	1.58E-03	7.11E-04	8.53E-04	0.00E+00	1.32E-05
505	2.36E-03	8.95E-04	1.71E-03	1.86E-03	4.77E-04	1.54E-03	0.00E+00	1.27E-05
510	2.57E-03	1.12E-03	1.88E-03	2.08E-03	3.38E-04	2.54E-03	1.36E-06	3.24E-06
515	2.72E-03	1.27E-03	1.89E-03	2.17E-03	2.63E-04	3.75E-03	3.71E-06	0.00E+00
520	2.83E-03	1.36E-03	1.83E-03	2.17E-03	2.21E-04	4.80E-03	2.65E-06	1.26E-06
525	2.90E-03	1.37E-03	1.73E-03	2.08E-03	1.81E-04	5.18E-03	1.20E-06	1.13E-06
530	2.96E-03	1.26E-03	1.60E-03	1.87E-03	1.62E-04	4.72E-03	0.00E+00	0.00E+00
535	3.02E-03	1.09E-03	1.44E-03	1.58E-03	1.46E-04	3.75E-03	0.00E+00	5.74E-07
540	3.06E-03	9.40E-04	1.31E-03	1.32E-03	1.24E-04	2.82E-03	2.51E-06	1.46E-05
545	3.10E-03	8.48E-04	1.22E-03	1.15E-03	1.37E-04	2.10E-03	1.20E-06	0.00E+00
550	3.12E-03	8.06E-04	1.16E-03	1.04E-03	1.23E-04	1.55E-03	2.59E-06	4.10E-06
555	3.12E-03	8.12E-04	1.12E-03	9.74E-04	1.19E-04	1.11E-03	8.26E-06	1.89E-06
560	3.12E-03	8.47E-04	1.10E-03	9.47E-04	1.08E-04	7.69E-04	1.54E-05	7.89E-06
565	3.10E-03	9.15E-04	1.10E-03	9.57E-04	1.15E-04	5.34E-04	2.78E-05	1.13E-06
570	3.06E-03	9.93E-04	1.10E-03	9.83E-04	1.16E-04	3.74E-04	5.42E-05	1.73E-05
575	3.01E-03	1.08E-03	1.10E-03	1.01E-03	9.38E-05	2.65E-04	1.02E-04	1.23E-05
580	2.95E-03	1.16E-03	1.11E-03	1.04E-03	1.10E-04	1.85E-04	1.94E-04	1.08E-05
585	2.90E-03	1.23E-03	1.13E-03	1.06E-03	1.02E-04	1.28E-04	3.64E-04	1.96E-05
590	2.83E-03	1.32E-03	1.14E-03	1.08E-03	1.07E-04	8.98E-05	6.72E-04	2.02E-05
595	2.77E-03	1.44E-03	1.18E-03	1.09E-03	1.05E-04	6.35E-05	1.22E-03	5.38E-05
600	2.69E-03	1.63E-03	1.23E-03	1.10E-03	8.92E-05	4.54E-05	2.17E-03	1.45E-04
605	2.60E-03	1.97E-03	1.35E-03	1.13E-03	7.59E-05	3.23E-05	3.82E-03	3.68E-04
610	2.49E-03	2.58E-03	1.55E-03	1.19E-03	8.50E-05	2.51E-05	6.67E-03	7.61E-04
615	2.37E-03	3.60E-03	1.89E-03	1.32E-03	9.97E-05	1.85E-05	1.11E-02	1.37E-03
620	2.24E-03	5.05E-03	2.37E-03	1.52E-03	7.55E-05	1.39E-05	1.67E-02	2.38E-03
625	2.10E-03	5.96E-03	2.61E-03	1.75E-03	8.57E-05	1.12E-05	1.81E-02	4.01E-03
630	1.95E-03	4.89E-03	2.13E-03	1.77E-03	7.83E-05	8.15E-06	9.26E-03	6.70E-03
635	1.81E-03	3.03E-03	1.52E-03	1.49E-03	8.13E-05	7.52E-06	3.20E-03	1.09E-02
640	1.66E-03	1.95E-03	1.20E-03	1.36E-03	8.33E-05	6.49E-06	1.11E-03	1.68E-02
645	1.51E-03	1.81E-03	1.17E-03	1.56E-03	8.61E-05	5.34E-06	3.95E-04	2.64E-02
650	1.37E-03	2.18E-03	1.32E-03	2.04E-03	7.81E-05	4.48E-06	1.61E-04	4.14E-02
655	1.23E-03	2.79E-03	1.56E-03	2.68E-03	7.16E-05	5.03E-06	7.69E-05	5.89E-02
660	1.10E-03	2.53E-03	1.41E-03	2.44E-03	9.32E-05	3.91E-06	3.60E-05	6.20E-02
665	9.75E-04	1.22E-03	8.26E-04	1.19E-03	7.54E-05	4.56E-06	2.27E-05	3.21E-02
670	8.59E-04	6.65E-04	5.56E-04	6.54E-04	7.85E-05	1.90E-06	1.54E-05	1.19E-02
675	7.55E-04	4.61E-04	4.37E-04	4.61E-04	5.75E-05	3.81E-06	3.50E-06	4.63E-03
680	6.58E-04	3.85E-04	3.71E-04	3.98E-04	5.30E-05	2.68E-06	1.01E-05	1.91E-03
685	5.75E-04	3.71E-04	3.27E-04	3.93E-04	9.03E-05	4.49E-06	6.64E-07	8.47E-04
690	5.03E-04	3.85E-04	2.95E-04	4.28E-04	7.69E-05	3.99E-06	3.66E-06	4.22E-04
695	4.37E-04	4.40E-04	2.72E-04	5.05E-04	7.92E-05	2.66E-06	0.00E+00	2.16E-04
700	3.75E-04	5.38E-04	2.59E-04	6.41E-04	6.32E-05	1.68E-07	2.35E-06	1.12E-04
705	3.27E-04	7.00E-04	2.55E-04	8.54E-04	4.58E-05	8.08E-07	5.69E-06	9.29E-05
710	2.77E-04	9.13E-04	2.62E-04	1.14E-03	1.00E-04	1.01E-06	9.61E-08	5.11E-05
715	2.35E-04	1.18E-03	2.78E-04	1.47E-03	9.45E-05	3.77E-06	9.68E-06	3.32E-05
720	2.05E-04	1.45E-03	2.97E-04	1.82E-03	7.46E-05	4.12E-06	8.51E-06	4.54E-05
725	1.72E-04	1.78E-03	3.27E-04	2.26E-03	1.31E-04	1.14E-06	1.04E-05	5.59E-05
730	1.51E-04	2.17E-03	3.64E-04	2.77E-03	1.23E-04	6.14E-06	7.71E-06	3.36E-05
735	1.27E-04	2.32E-03	3.72E-04	2.96E-03	7.77E-05	3.33E-06	0.00E+00	2.62E-05
740	1.11E-04	1.68E-03	2.82E-04	2.14E-03	1.20E-04	4.36E-06	8.44E-06	4.46E-05
745	9.63E-05	8.17E-04	1.62E-04	1.02E-03	1.24E-04	5.50E-06	1.27E-05	3.51E-05
750	8.42E-05	3.72E-04	1.00E-04	4.49E-04	1.72E-04	5.18E-06	1.45E-05	7.30E-08
755	7.10E-05	1.85E-04	7.20E-05	2.20E-04	2.25E-04	1.33E-05	1.54E-05	1.07E-04
760	6.29E-05	1.18E-04	6.06E-05	1.20E-04	1.97E-04	1.95E-05	1.92E-05	6.25E-05
765	4.61E-05	8.51E-05	5.61E-05	7.98E-05	1.96E-04	3.03E-05	3.75E-05	1.25E-05
770	4.27E-05	7.72E-05	6.27E-05	6.76E-05	4.07E-04	4.17E-05	3.43E-05	6.62E-05
775	3.14E-05	8.11E-05	7.02E-05	7.11E-05	2.51E-04	4.46E-05	5.21E-05	1.65E-04
780	3.78E-05	9.46E-05	8.75E-05	6.89E-05	7.03E-04	7.09E-05	9.28E-05	1.47E-04
